# Enhancing Cognitive Functions and Neuronal Growth through NPY1R Agonist and Ketamine Co-Administration: Evidence for NPY1R-TrkB Heteroreceptor Complexes in Rats

**DOI:** 10.3390/cells13080669

**Published:** 2024-04-12

**Authors:** Carlos Arrabal-Gómez, Rasiel Beltran-Casanueva, Aracelis Hernández-García, Juan Vicente Bayolo-Guanche, Miguel Angel Barbancho-Fernández, Pedro Jesús Serrano-Castro, Manuel Narváez

**Affiliations:** 1 NeuronLab, Facultad de Medicina, Instituto de Investigación Biomédica de Málaga, Universidad de Málaga, 29071 Málaga, Spain; carrabal@uma.es (C.A.-G.); mabarbancho@uma.es (M.A.B.-F.); 2 Facultad de Psicología, Instituto de Investigación Biomédica de Málaga, Universidad de Málaga, 29071 Málaga, Spain; 3 Unit of Neurology, Hospital Regional Universitario de Málaga, Instituto de Investigación Biomédica de Málaga, 29010 Málaga, Spain; 4 Vithas Málaga, Grupo Hospitalario Vithas, 29016 Málaga, Spain; 5 Department of Neuroscience, Karolinska Institutet, 17177 Stockholm, Sweden; verdeverde1988@gmail.com (R.B.-C.); aracelishernandezgarcia81@gmail.com (A.H.-G.); bayolo1969@gmail.com (J.V.B.-G.); 6 Receptomics and Brain Disorders Lab, Edificio Lopez-Peñalver, Instituto de Investigación Biomédica de Málaga, Universidad de Málaga, 29071 Málaga, Spain

**Keywords:** NPY1R agonist, ketamine, cognitive functions, neuronal proliferation, NPY1R-TrkB heteroreceptor complex, brain-derived neurotrophic factor (BDNF), neurodegenerative diseases, dorsal hippocampus, memory consolidation, neuropharmacology

## Abstract

This study investigates the combined effects of the neuropeptide Y Y1 receptor (NPY1R) agonist [Leu31-Pro34]NPY at a dose of 132 µg and Ketamine at 10 mg/Kg on cognitive functions and neuronal proliferation, against a backdrop where neurodegenerative diseases present an escalating challenge to global health systems. Utilizing male *Sprague-Dawley* rats in a physiological model, this research employed a single-dose administration of these compounds and assessed their impact 24 h after treatment on object-in-place memory tasks, alongside cellular proliferation within the dorsal hippocampus dentate gyrus. Methods such as the in situ proximity ligation assay and immunohistochemistry for proliferating a cell nuclear antigen (PCNA) and doublecortin (DCX) were utilized. The results demonstrated that co-administration significantly enhanced memory consolidation and increased neuronal proliferation, specifically neuroblasts, without affecting quiescent neural progenitors and astrocytes. These effects were mediated by the potential formation of NPY1R-TrkB heteroreceptor complexes, as suggested by receptor co-localization studies, although further investigation is required to conclusively prove this interaction. The findings also highlighted the pivotal role of brain-derived neurotrophic factor (BDNF) in mediating these effects. In conclusion, this study presents a promising avenue for enhancing cognitive functions and neuronal proliferation through the synergistic action of the NPY1R agonist and Ketamine, potentially via NPY1R-TrkB heteroreceptor complex formation, offering new insights into therapeutic strategies for neurodegenerative diseases.

## 1. Introduction

Neurodegenerative disorders, including Alzheimer’s disease, Huntington’s disease, Parkinson’s disease, and amyotrophic lateral sclerosis (ALS), constitute a critical area of neurological research due to their chronic nature, incurability, and profound impact on individuals and society. The progressive degeneration of neuronal populations characterizes these conditions, leading to severe disability. The recent COVID-19 pandemic has further highlighted the intersection between infectious diseases and neurological health, with emerging evidence suggesting an exacerbation of cognitive and psychiatric symptoms post-infection, underscoring the need for broadened research into brain health and disease [1,2]. Furthermore, neurodegenerative diseases have been shown to attenuate and impair adult hippocampal neurogenesis (AHN), significantly influencing the progression and manifestation of these conditions [3,4,5,6,7].

AHN is essential for neuronal plasticity, supporting learning, memory, and emotional regulation under normal physiological conditions [8,9,10]. This process is pivotal for learning, the acquisition and consolidation of memory, and pattern separation, crucial for differentiating between similar experiences [11,12,13,14,15].

Brain-derived neurotrophic factor (BDNF) plays a significant role in supporting AHN, with drugs like Memantine and Donepezil affecting BDNF levels and demonstrating potential therapeutic effects, despite their limited efficacy in addressing depressive symptoms [16,17,18,19]. Memantine is not able to block calcium entry via a blockade of NMDA receptors, while Ketamine does [20,21,22]. Conversely, Ketamine’s ability to block calcium entry via NMDA receptors and its effects on extracellular glutamate levels, AMPA receptor activation, and BDNF release offers insight into novel avenues for cognitive enhancement and neurogenesis, especially given its direct binding to the TrkB receptor [23,24,25,26].

Moreover, neuropeptides such as neuropeptide Y (NPY) have been associated with the cellular and molecular mechanisms underlying AHN and cognitive functionalities [27,28,29,30]. Studies have demonstrated NPY’s role in promoting neurogenesis within hippocampal stem cells, significantly in both in vitro and in vivo contexts [31,32]. Enhancements in hippocampal NPY mRNA expression following spatial learning tasks have been observed in rats, though aging rats display a correlation between reduced NPY expression in the hippocampus and diminished memory and neurogenesis [33,34]. Thus, the therapeutic potential of targeting NPY Y1 receptors (NPY1Rs) for improving dentate neurogenesis, cognitive performance, and neuronal health spatial learning through increasing BDNF has been highlighted [35,36,37,38,39]. Our study aims to explore the synergistic effects of an NPY1R agonist and Ketamine on BDNF expression, neuronal growth, and cognitive enhancement, focusing on their potential to modulate AHN and its underlying mechanisms.

Given this background, our study specifically examines the synergistic effects of the NPY1R agonist and Ketamine administration on BDNF expression and its consequential impact on neuronal growth and cognitive enhancement. This research not only elucidates the potential of NPY1R-TrkB heteroreceptor complexes as a novel therapeutic target for neurodegenerative diseases but also seeks to deepen our understanding of the mechanisms behind adult hippocampal neurogenesis (AHN), particularly in relation to cognitive performance and neuronal proliferation in the dorsal hippocampus. By investigating the roles of BDNF and the formation of NPY1R-TrkB heteroreceptor complexes, we aim to provide a comprehensive analysis of the underlying mechanisms of these effects. Crucially, this study is designed to augment the current corpus of knowledge concerning the management of neurodegenerative diseases, thereby paving the way for the development of innovative therapeutic strategies. These strategies are anticipated to harness the neurogenic and cognitive potential of pharmacological interventions targeting the NPY1R and Ketamine pathways, offering significant promise for enhancing patient outcomes in neurodegenerative disease contexts.

## 2. Materials and Methods

### 2.1. Animals

We selected male *Sprague-Dawley* rats aged 6–8 weeks and weighing 200–250 g for this study, supplied by CRIFFA, Barcelona, Spain. These animals had unrestricted access to food and water and were housed under a controlled environment featuring a 12 h light/dark cycle, with the temperature and relative humidity maintained at 22 ± 2 °C and 55–60%, respectively. The experimental protocols employed were sanctioned by the University of Málaga’s Local Ethics Committee for Animal Care and Use (CEUMA 45-2022-A), adhering to the guidelines of the EU Directive 2010/63/EU and Spain’s Real Decretory 53/2013.

### 2.2. Preparation of Drugs

The pharmacological agents for this research were sourced as follows: the NPY1R agonist [Leu31, Pro34]NPY and the NPY1R antagonist BIBP3226 were acquired from Tocris Bioscience, UK, while the TrkB antagonist ANA-12 was obtained from Sigma Aldrich, MO, USA. Ketamine (Ketolar, Pfizer, Freiburg, Germany) was procured from the University of Málaga’s animal facility, Spain. Detailed procedures for the intranasal administration are provided in the Supplementary Documents.

### 2.3. Behavioral Analysis

#### Assessment of Spatial Memory in Rats

The object-in-place task was chosen for its minimal stress impact on subjects and utilized as a measure of spatial recognition memory, assessing the rats’ ability to spontaneously explore objects. This task is recognized for its significant translational value, bridging rodent behavioral research and cognitive assessments relevant to human neurological conditions. The task’s design, wherein the choice trial is conducted 24 h following the sample trial, is specifically intended to evaluate natural forgetting processes, thus providing insights into the mechanisms supporting sustained memory retention. Such an approach not only facilitates the assessment of spatial memory capabilities in rats but also reflects the task’s potential applicability in exploring memory dynamics [40,41].

The intranasal administration of peptides and Ketamine was performed 24 h prior to testing, with each administration comprising a total volume of 20 μL. The selection of experimental groups and the dosing regimen for Ketamine and peptides—including the 10 mg/kg dose for Ketamine—were based on established efficacy parameters from prior research, particularly focusing on the effective antidepressant-like doses and their impact on cognitive functions [42,43,44,45,46,47,48]. This approach was guided by dose–response studies that have identified the optimal dosage to explore the neuropharmacological effects of Ketamine, including its complex influence on memory processes, without significantly affecting spatial memory consolidation. Animals were randomly divided: (1) control, using distilled water; (2) NPY1R-agonist-treated group, receiving [Leu31-Pro34]NPY (132 µg); (3) Ketamine-treated group, receiving Ketamine (10 mg/Kg); (4) a group administered both the NPY1R agonist and Ketamine; (5) a group co-administered the NPY1R agonist, Ketamine, and the NPY1R antagonist BIBP3226 (132 µg); and (6) a group treated with the NPY1R agonist, Ketamine, and the TrkB antagonist ANA-12 (0.5 mg/kg). In the context of our experimental design, both ANA-12 and BIBP3226 did not exhibit effects on memory per se, suggesting that these compounds, when administered, do not significantly influence memory functions [49,50].

**Figure 1:**
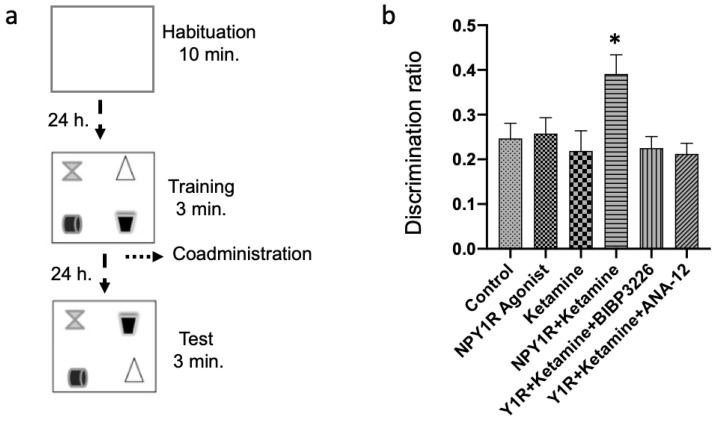
Evaluating spatial memory following the combined intranasal administration of the NPY1R agonist and Ketamine in the object-in-place memory task. (**a**) A schematic representation of the sequential phases in the object-in-place memory task, spaced 24 h apart. The habituation phase allows for free exploration without objects for ten minutes. The training phase then introduces four different objects for a three-minute exploration period. Finally, the test phase involves a three-minute exploration with two of the original objects switched in position. (**b**) Performance metrics for the object-in-place task, demonstrating the capacity of rats to discern the switched objects after three weeks of intranasal infusion with the NPY1R agonist and Ketamine. Notably, the co-administration of NPY1R (Y1R receptor agonist [Leu31-Pro34]NPY, 132 µg) and Ketamine (10 mg/kg) resulted in improved performance on the object-in-place task. However, this enhancement was counteracted by the addition of BIBP3226 (NPY1R antagonist, 132 µg) or ANA-12 (TrkB antagonist, 0,5 mg/kg). Data are expressed as the mean ± SEM of the discrimination ratio during the test phase (*n* = 6 animals per group). * *p* < 0.05, indicating a significant difference compared to the rest of the groups, as determined by the one-way ANOVA and post hoc Newman–Keuls test. Abbreviations: Control = distilled water; NPY1R Agonist = neuropeptide Y1 receptor agonist [Leu31, Pro34]NPY (132 µg); Ketamine = administration of Ketamine (10 mg/kg); NPY1R + Ketamine = co-administration of NPY1R agonist and Ketamine; NPY1R + Ketamine + BIBP3226 = co-administration of NPY1R agonist, Ketamine, and NPY1R antagonist BIBP3226 (132 µg); NPY1R + Ketamine + ANA-12 = co-administration of NPY1R agonist, Ketamine, and TrkB antagonist ANA-12 (0.5 mg/kg).

Behavioral experiments were conducted between 09:00 and 14:00 h to minimize variability related to diurnal rhythms. Animals were acclimatized to handling and the experimental room (80–90 lux) for at least 1 h before testing to ensure proper habituation. During the behavioral assessment period, rats were single-housed to prevent social influences on stress levels and cognitive performance. The object-in-place memory task consisted of three phases—habituation, training, and testing—carried out as per established protocols (Figure 1a) [42,51,52,53]. During the habituation phase, animals were handled for two days to reduce stress associated with experimenter interaction and then familiarized with an empty arena (100 × 100 × 60 cm) for 10 min in one trial to adapt to the testing environment. In the training phase, each rat was placed in the center of the arena, 24 h following habituation, and allowed to explore four distinct objects placed at the corners of the arena for 3 min. These objects, differing in color and shape but similar in weight and size, were positioned 10 cm from the sidewalls and were cleaned with 5% ethanol after each trial to eliminate olfactory cues. The testing phase, conducted 24 h post-training, involved exchanging two of the objects to assess the rats’ discrimination ability. The discrimination index (DI) was calculated based on the time spent exploring the newly positioned objects versus those that remained in their original location, with a higher DI indicating intact object-in-place memory, implying an improvement in spatial memory. Care was taken to counterbalance object locations between trials and rats, and the arena and objects were thoroughly cleaned with 5% ethanol between sessions to ensure no residual scent cues.

### 2.4. Cellular and Molecular Analysis

A different cohort of rats was used for the subsequent procedures. Rats were randomly distributed into five experimental groups—(1) control: distilled water; (2) NPY1R-agonist-treated group receiving the NPY1R agonist [Leu^31^-Pro^34^]NPY (132 µg); (3) Ketamine-treated group receiving the Ketamine (10 mg/Kg); (4) NPY1R+Ketamine: group administered with both substances; (5) NPY1R+Ketamine+ANA-12: group treated with [Leu^31^-Pro^34^]NPY, Ketamine, and the TrkB antagonist (ANA-12; 0,5 mg/kg µg) (n = 4 in each group). A total of 24 h after the administration of the treatments, the sequence of cellular and molecular analyses was initiated, and the rats were deeply anesthetized with pentobarbital (Mebumal, 100 mg/kg, i.p.) and perfused transcardially with 4% PFA (paraformaldehyde, wt./vol, Sigma Aldrich, St. Louis, MI, USA). The brain tissues were precisely sectioned at a 30 μm thickness across the dorsal hippocampus (posterior in primates) (from −1.60 to −5.30 Bregma; Paxinos and Watson, 2006 [54]).

#### 2.4.1. In Situ Proximity Ligation Assay

To assess the co-localization of NPY1R and TrkB within the dorsal dentate gyrus, we employed the in situ proximity ligation assay technique using NaveniFlex Tissue GR Atto 647N probes (Navinci, Sweden), following a method adapted from previously described protocols [55,56]. The analysis began with the preparation of brain tissue sections, which were submerged in a blocking buffer at 37 °C for one hour within a humidity-controlled chamber to prevent non-specific binding. This step was succeeded by an overnight antibody incubation at 4 °C, utilizing specifically goat anti-NPY1R (1:200, sc-21992, Santa Cruz Biotechnology, Santa Cruz, CA, USA) and rabbit anti-TrkB (1:200, Sigma Aldrich, St. Louis, MO, USA, ZRB1281) antibodies. After the incubation period, sections were thoroughly washed and then incubated with a prepared mix of goat and rabbit Navenibodies for one hour at 37 °C. Following additional washes to remove unbound probes, the sections were sequentially exposed to Enzymes A and B within a humidity chamber at 37 °C, for durations of 60 min and 30 min, respectively. Excess oligonucleotides were washed off before applying the rolling circle amplification detection mix (Enzyme C, Tex615) for a 90 min incubation at 37 °C. Finally, the sections were mounted using a DAPI-containing fluorescent mounting medium (Abcam, Cambridge, UK, ab104139), preserved at −20 °C, and subjected to a confocal microscopy analysis according to the outlined references [42,45,57].

#### 2.4.2. Evaluation of Hippocampal Cell Proliferation

We conducted an immunohistochemical analysis starting with antigen retrieval, which involved heating the tissue sections in a saline sodium citrate buffer (pH 6; 10 mM sodium citrate) at 65 °C for 90 min to unmask antigens. Following this, sections were submerged in a 0.6% hydrogen peroxide solution for 30 min to quench endogenous peroxidase activity, essential for reducing background staining. Next, the sections underwent overnight incubation at 4 °C with a primary antibody against PCNA (P8825, Sigma Aldrich, MO, USA, 1:1500), aimed at identifying proliferating cells. This was followed by a 90 min incubation at room temperature with a biotinylated anti-mouse IgG secondary antibody (Vector Laboratories, Newark, CA, USA, 1:200) to enable signal detection. Signal amplification was achieved using ExtrAvidin peroxidase (Sigma Aldrich, MO, USA, 1:100) in a dimly lit setting for 60 min, with the PCNA signal subsequently visualized through the application of a 0.05% diaminobenzidine and 0.03% H_2_O_2_ solution in PBS. After thorough washing, sections were mounted, dehydrated, and cover-slipped in preparation for microscopy. The enumeration of PCNA-positive cells was conducted utilizing the optical fractionator method within an unbiased stereology framework, employing an Olympus BX51 microscope [44,53,58,59]. This procedure is further elaborated in our Supplementary Methods.

#### 2.4.3. Double Immunofluorescence Protocol

For cell lineage tracing, double immunofluorescence was executed to identify the phenotypes of proliferating cells [42,53,56,60,61]. This procedure involved initial blocking and permeabilization with 5% goat serum and 0.3% Triton X-100 in PBS for 60 min each. The sections were then incubated with combinations of primary antibodies, mouse anti-PCNA (Sigma, P8825, 1:1500) with rabbit anti-DCX (Abcam, Cambridge, UK, ab18723, 1:2000) or mouse anti-PCNA with rabbit anti-GFAP (Abcam, Cambridge, UK, ab7260, 1:1500), for 24 h at 4 °C. Appropriate secondary antibodies, donkey anti-mouse AlexaFluor 488 and donkey anti-rabbit AlexaFluor 647 (both from Abcam, Cambridge, UK, 1:200), were applied thereafter. Sections were finally mounted using a DAPI-containing fluorescent medium (Abcam, Cambridge, UK, ab104139). A quantitative analysis of double-labeled cells within the dentate gyrus was carried out as previously established [53,62].

#### 2.4.4. Brain-Derived Neurotrophic Factor (BDNF) Induction Evaluation

To determine BDNF induction in the dorsal dentate gyrus, we processed separate brain sections with antigen retrieval and peroxidase neutralization steps similar to those described above. The sections were incubated with a primary BDNF antibody (Chemicon, Sigma Aldrich, MO, USA, AB1534SP, 1:500, in 2.5% donkey serum) at room temperature. Following PBS washes, the application of a biotinylated anti-rabbit IgG secondary antibody (Sigma Aldrich, MO, USA, B8895, 1:200) and ExtrAvidin peroxidase (Sigma Aldrich, MO, USA, 1:100) was performed in darkness. The visualization of BDNF-expressing cells used a DAB and H2O2 mixture. Prepared sections were mounted, cover-slipped, and analyzed for BDNF-positive cells using the optical fractionator method with unbiased stereology on an Olympus BX51 microscope.

### 2.5. Statistical Analysis

Data are presented as the mean ± SEM, with sample sizes detailed in the figure legends. The statistical analysis was conducted using GraphPad PRISM 8.0 software. The selection of statistical tests was predicated on the data distribution and the experimental design. A preliminary assessment of normality was performed for all datasets to determine the appropriate statistical tests.

For the object-in-place memory task, comparing six groups (n = 6 per group), a one-way ANOVA followed by a post hoc Newman–Keuls test was employed to evaluate differences among the groups, chosen for its robustness in handling multiple comparisons while controlling the Type I error rate. In assessing NPY1R/TrkB heteroreceptor complex formation, PCNA labeling, and BDNF expression where five groups were compared (n = 4 per group), a one-way ANOVA with a Newman–Keuls post hoc test was utilized to discern significant differences between groups, due to its efficacy in multiple group comparisons and its ability to conduct pairwise comparisons post-ANOVA. For the comparison of DCX+/PCNA+ cells and DCX+/PCNA- cells, the Student’s unpaired *t*-test was used (n = 4), as this test is suitable for comparing means between two independent groups when data are normally distributed. For datasets not meeting the normality assumption, alternative non-parametric tests were considered, though all analyzed data conformed to normal distribution criteria, allowing for the use of the aforementioned parametric tests. Significance levels were set at * *p* < 0.05, ** *p* < 0.01, and *** *p* < 0.001, reflecting standard thresholds for statistical significance.

## 3. Results

### 3.1. Improvement in Spatial Memory through NPY1R Agonist Infusion and Ketamine

Following the administration of treatments, spatial memory was assessed using the object-in-place task 24 h later. This task encompassed a habituation stage, allowing rats 10 min of object-free exploration, followed by a training phase with four distinct objects. In the subsequent test phase, the spatial arrangement of two objects was altered to evaluate memory retention (illustrated in Figure 1a).

Remarkably, the combined intranasal administration of an NPY1R agonist with Ketamine after the acquisition phase significantly bolstered object-in-place memory consolidation compared to control and other treatment groups (one-way ANOVA, F5, 30 = 3.62, *p* < 0.05; Newman–Keuls post hoc test: *p* < 0.05, depicted in Figure 1b). The beneficial memory effects were mitigated upon introducing BIBP3226, an NPY1R antagonist, underscoring its potential in modulating memory (post hoc test results: *p* < 0.05). Neither the sole administration of the NPY1R agonist nor Ketamine displayed significant impacts on memory tasks (Figure 1b), aligning with control observations. Crucially, the role of BDNF in enhancing memory was substantiated by administering ANA-12, a TrkB antagonist, which negated the combined effect of the NPY1R agonist and Ketamine on memory consolidation (Newman–Keuls post hoc test: *p* < 0.05; Figure 1b).

Additionally, evaluations of total exploration time in both training and test phases revealed no notable changes in exploratory behavior or spontaneous motor activity post-treatment, indicating the treatments’ specificity to cognitive functions without affecting general activity levels (Supplementary Table S1). Furthermore, the time spent exploring the exchanged objects was significantly elevated in the NPY1R+Ketamine group relative to control (one-way ANOVA, F5, 30 = 5.92, *p* < 0.001; Newman–Keuls post hoc test: *p* < 0.01), NPY1R agonist (Newman–Keuls post hoc test: *p* < 0.01), Ketamine (Newman–Keuls post hoc test: *p* < 0.001), Y1R+Ketamine+BIBP3226 (Newman–Keuls post hoc test: *p* < 0.01), and Y1R+Ketamine+ANA-12 (Newman–Keuls post hoc test: *p* < 0.01) groups.

### 3.2. NPY1R and TrkB Co-Activation Promotes Heteroreceptor Complex Formation

**Figure 2:**
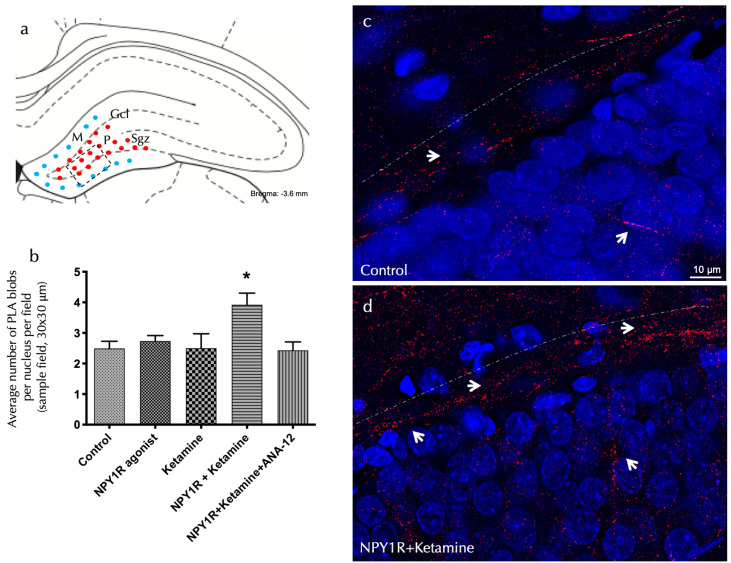
The visualization of NPY1R/TrkB heteroreceptor complex formation in the dorsal hippocampal DG via a proximity ligation assay. The identification of NPY1R/TrkB heteroreceptor complexes within the dorsal hippocampal dentate gyrus (DG) through the application of the in situ proximity ligation assay (PLA). This innovative technique allows for the direct observation of protein–protein interactions in close proximity, utilizing highly specific primary antibodies targeted at each protein. The assay highlights the following: (**a**) The presence of PLA-positive signals (shown as red dots) primarily within the subgranular zone (SGZ) of the DG, delineating the interface between the granule cell layer (GCL) and the polymorphic layer (PL) in the dorsal hippocampus. These signals extend into the PL, with the molecular layer (ML) characterized by the absence of PLA signals (indicated by blue dots). The anatomical landmarks correspond to the Bregma −3.6 mm coordinate according to the Paxinos and Watson [54] stereotaxic atlas. (**b**) A quantitative analysis of PLA signals in the SGZ involved counting the red PLA-positive spots per nucleus in each field, carried out by an analyst blind to the experimental conditions. (**c**,**d**) Representative images showcase a marked elevation in the density of red PLA blobs indicative of NPY1R/TrkB heteroreceptor complexes in the SGZ following the co-administration of the NPY1R agonist and Ketamine, relative to the control group. These complexes are depicted as red PLA blobs (clusters) with high densities per cell across numerous neurons, visualized using confocal laser microscopy. Statistical annotations include * *p* < 0.05, indicating a significant difference compared to the rest of the groups, as determined by the one-way ANOVA and Newman–Keuls post hoc test (*n* = 4). Vertical lines above bars facilitate inter-group comparisons. Data are presented as the mean ± SEM for four rats per group, in duplicates. In our visual representations, white arrows highlight the presence of PLA-positive clusters, indicative of protein interactions detected by the proximity ligation assay. Dashed lines demarcate the granule cell layer (GCL) of the dentate gyrus (DG), providing a structural reference for the observed molecular interactions. Additionally, nuclei have been counterstained in blue with DAPI, offering contrast and clarity to the cellular components within the tissue sections. Abbreviations: Control = distilled water; NPY1R agonist = neuropeptide Y1 receptor agonist [Leu31, Pro34]NPY (132 µg); Ketamine = administration of Ketamine (10 mg/kg); NPY1R+Ketamine = co-administration of NPY1R agonist and Ketamine; NPY1R+Ketamine+BIBP3226 = co-administration of NPY1R agonist, Ketamine, and NPY1R antagonist BIBP3226 (132 µg); NPY1R+Ketamine+ANA-12 = co-administration of NPY1R agonist, Ketamine, and TrkB antagonist ANA-12 (0.5 mg/kg).

Investigating the receptor-level mechanisms behind the observed cognitive effects entailed using an in situ proximity ligation assay (PLA) within the dorsal dentate gyrus (DG). This method allowed for the detection of NPY1R/TrkB heteroreceptor complex formation, which was significantly enhanced following the co-administration of the NPY1R agonist and Ketamine. The assay identified marked increases in positive red cluster density within the dorsal DG’s subgranular and polymorphic layers (one-way ANOVA, F4, 20 = 3.76, *p* < 0.05), with post hoc testing confirming these findings (*p* < 0.05 against control and other conditions), as showcased in Figure 2a,d.

The inhibitory influence of ANA-12 on these effects, demonstrated through the post hoc analysis (*p* < 0.05), underscores TrkB’s pivotal role in mediating these receptor interactions. Figure 2 illustrates these PLA-positive red clusters in the dorsal DG, with subpanels elucidating cluster density variations across treatment conditions. Importantly, neither the NPY1R agonist nor Ketamine alone significantly altered PLA-positive red cluster densities in the dorsal hippocampus, as detailed in Figure 2b.

### 3.3. Enhanced Cell Proliferation in the Dorsal Hippocampus through NPY1R Agonist and Ketamine Combination

**Figure 3:**
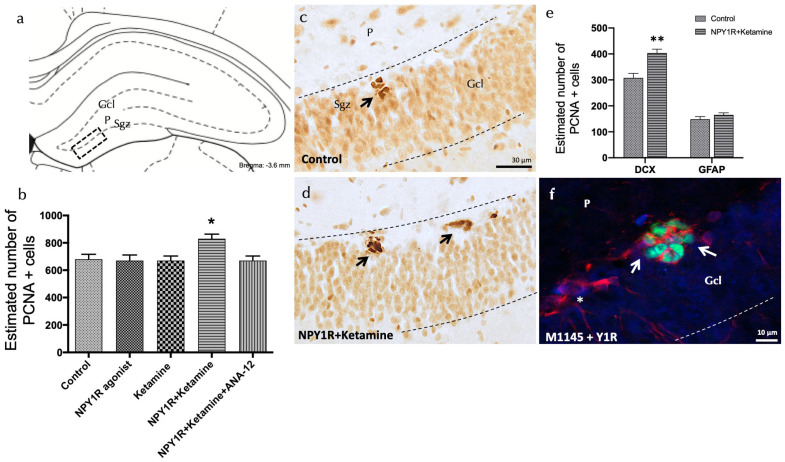
Enhancing neurogenesis in the dorsal dentate gyrus through the intranasal co-administration of the NPY1R agonist and Ketamine. Impact of co-administering an NPY1R agonist and Ketamine on neurogenesis within the dorsal dentate gyrus in adult rats, as evidenced by PCNA immunostaining. This analysis compares the effects of administering the NPY1R receptor agonist and Ketamine, both individually and jointly, with and without the presence of the TrkB receptor antagonist ANA-12. (**a**,**d**) Predominantly, PCNA-positive cells localize to the subgranular zone (SGZ) at the boundary of the granule cell layer (GCL) and the polymorphic layer (P) within the dorsal hippocampus, often forming clusters. This area corresponds to the Bregma −3.6 mm location as per the Paxinos and Watson stereotaxic guide [54]. (**b**) presents a quantitative analysis of PCNA-immunoreactive (IR) cells within the SGZ, showing differences across groups treated with control conditions, the NPY1R agonist [Leu31-Pro34]NPY, Ketamine, or their co-administration with or without ANA-12. (**d**) highlights the increased PCNA labeling in the SGZ following the combined treatment compared to control conditions (**c**), with arrows pointing to clusters of PCNA-positive neurons and dashed lines outlining the GCL. (**e**) quantifies PCNA-IR cells that are also double-labeled with DCX, indicating that the co-administration specifically targets neuroblast proliferation. ** *p* <0.01 indicates significant enhancement versus the control, analyzed using Student’s *t*-test. (**f**) A representative image showcases DCX+/PCNA+ cells (white arrows) and DCX+/PCNA- cells (white asterisks) in the group receiving both the NPY1R agonist and Ketamine. Statistical significance is marked as * *p* < 0.05 versus the rest of the groups, determined through the one-way ANOVA and Newman–Keuls post hoc analysis (n = 4). Vertical and horizontal lines above the bars enable comparison between groups. Each experimental group consisted of four animals. Abbreviations: Control = distilled water; NPY1R agonist = neuropeptide Y1 receptor agonist [Leu31, Pro34]NPY (132 µg); Ketamine = administration of Ketamine (10 mg/kg); NPY1R+Ketamine = co-administration of NPY1R agonist and Ketamine; NPY1R + Ketamine + BIBP3226 = co-administration of NPY1R agonist, Ketamine, and NPY1R antagonist BIBP3226 (132 µg); NPY1R + Ketamine + ANA-12 = co-administration of NPY1R agonist, Ketamine, and TrkB antagonist ANA-12 (0.5 mg/kg).

Using proliferating cell nuclear antigen (PCNA) staining, we investigated the combined effect of the NPY1R agonist and Ketamine on cell proliferation within the adult dorsal hippocampus, focusing specifically on the subgranular zone (SGZ) of the dentate gyrus. A significant increase in cell proliferation was observed with the co-administration of these compounds, as evidenced by the heightened presence of PCNA-immunoreactive (PCNA-IR) cells in comparison to control and other experimental groups (one-way ANOVA, F4, 15 = 3.81, *p* < 0.05; Newman–Keuls post hoc test: *p* < 0.05) (Figure 3a,b,d).

The addition of the TrkB antagonist, ANA-12, counteracted the effects seen with the NPY1R agonist and Ketamine, underscoring TrkB’s vital role in facilitating cell proliferation through this drug interaction (Newman–Keuls post hoc test: *p* < 0.05) (Figure 3b). Neither the NPY1R agonist nor Ketamine alone induced significant changes in PCNA-IR cell counts within the SGZ, mirroring the findings with the control group (Figure 3a–c).

#### Determining the Cellular Types Influenced by Increased Proliferation

Further investigations aimed to identify the specific cell types affected by the proliferation induced by NPY1R agonist and Ketamine treatment. This was achieved by quantifying PCNA-labeled cells to detect co-expression with either doublecortin (DCX), indicative of neuroblasts, or Glial Fibrillary Acidic Protein (GFAP), indicative of dormant radial stem cells (Figure 3e). Notably, there was a significant rise in the number of PCNA+/DCX+ cells following treatment, compared to control subjects (*t* = 4.114, df = 6; *p* < 0.05) (Figure 3f), while the population of PCNA+/GFAP+ cells remained unchanged (*t* = 1.254, df = 6; *p* > 0.05). These results suggest that NPY1R agonist and Ketamine co-administration preferentially promotes the proliferation of neuroblasts without affecting dormant radial stem cells.

### 3.4. Correlation of Neuroblast Increase with BDNF Elevation via NPY1R Agonist and Ketamine Co-Activation

**Figure 4:**
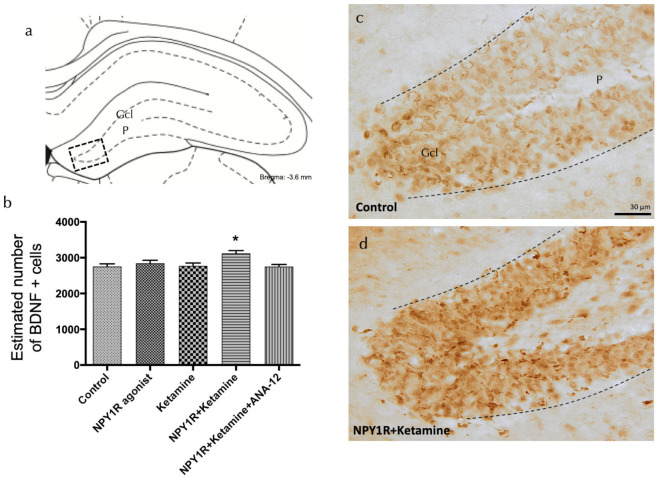
Enhancing BDNF expression in the dorsal dentate gyrus through the co-administration of the NPY1R agonist and Ketamine. Effects of co-administering an NPY1R agonist and Ketamine on the distribution of brain-derived neurotrophic factor immunoreactive (BDNF-IR) cells within the dorsal dentate gyrus (DG) of the hippocampal region. Panel (**a**) highlights that BDNF-IR cells are predominantly found in the granule cell layer (GCL) of the DG, with a sparse presence in the polymorphic layer (P), corresponding to the Bregma −3.6 mm location as per the Paxinos and Watson stereotaxic atlas [54]. Panel (**b**) quantitatively demonstrates a significant upsurge in BDNF-IR cells in the dorsal DG following the co-administration of the NPY1R agonist and Ketamine. The application of the TrkB antagonist ANA-12 is shown to counter this effect. Statistical significance is indicated by * *p* < 0.05 compared to the rest of the groups, as established by the one-way ANOVA and subsequent Newman–Keuls post hoc testing (*n* = 4 per group). The graph’s vertical lines above bars facilitate inter-group comparison. Data are depicted as the mean ± SEM. Panels (**c**,**d**) provide representative images showcasing the notable enhancement in BDNF-positive cells in the DG after the co-administration of the NPY1R agonist and Ketamine (**d**), relative to the control group treated with distilled water (**c**). Dashed lines mark the GCL boundaries within the DG. Abbreviations: Control = distilled water; NPY1R agonist = neuropeptide Y1 receptor agonist [Leu31, Pro34]NPY (132 µg); Ketamine = administration of Ketamine (10 mg/kg); NPY1R + Ketamine = co-administration of NPY1R agonist and Ketamine; NPY1R + Ketamine + BIBP3226 = co-administration of NPY1R agonist, Ketamine, and NPY1R antagonist BIBP3226 (132 µg); NPY1R + Ketamine + ANA-12 = co-administration of NPY1R agonist, Ketamine, and TrkB antagonist ANA-12 (0.5 mg/kg).

To elucidate the mechanisms driving the observed proliferation boost, we evaluated the expression of brain-derived neurotrophic factor (BDNF) in the dorsal hippocampal dentate gyrus following treatment with the NPY1R agonist, Ketamine, or their combination. BDNF-positive cells were predominantly observed in the granular layer and, to a lesser extent, in the polymorphic layer of the dorsal DG (Figure 4a). A significant increase in BDNF-positive cells was quantified following the combined drug treatment compared to all other groups (one-way ANOVA, F4, 15 = 4.35, *p* < 0.05; Newman–Keuls post hoc test: *p* < 0.05), as depicted in Figure 4a,d. The application of the TrkB antagonist ANA-12 nullified the observed increase in BDNF expression induced by the drug combination (Newman–Keuls post hoc test: *p* < 0.001) in Figure 4b, reinforcing the crucial involvement of TrkB in mediating the proliferative and neurotrophic response. In contrast, when administered independently, neither the NPY1R agonist nor Ketamine significantly impacted the level of BDNF-positive cells in the dorsal DG, emphasizing the unique synergistic effect of their combined administration.

## 4. Discussion

Our investigation has revealed significant insights into the combined neuropharmacological effects of the intranasal administration of an NPY1R agonist with Ketamine, revealing an enhancement in object-in-place memory performance and neuronal proliferation within the dentate gyrus of the dorsal hippocampus. This study, performed within a physiological context using healthy rats, points towards potential applications for improving cognitive functions and promoting neuronal growth. While our study focused on evaluating memory performance with a 24 h intertrial interval to assess sustained memory retention, we acknowledge the value of exploring shorter intertrial intervals, such as 1–2 h, to provide a comparative analysis of memory consolidation processes. Future studies could benefit from incorporating such assessments to elucidate the temporal dynamics of memory performance in normal rats, potentially revealing variations in their ability to consolidate memory over shorter versus longer intervals.

While the singular administration of an NPY1R agonist or Ketamine did not produce significant results in our object-in-place task, the literature suggests that NPY and its agonists have the capacity to ameliorate spatial memory deficits in rodent models mimicking Alzheimer’s disease symptoms [63]. Additionally, studies have shown a decrease in NPY-immunoreactive fibers within the dentate gyrus in Alzheimer’s disease models, indicating the potential of NPY1R agonists in modulating hippocampal functions [63]. The importance of NPY1R signaling in cognitive functions has been further emphasized by research on conditional mice with hippocampal-specific Npy1r gene inactivation, which revealed impaired spatial learning [64]. These findings, together with comparative studies suggesting species-specific responses [65,66,67], highlight the complex nature of NPY1R and Ketamine’s impact on cognitive processes. Ketamine’s lack of impact on the object-in-place task, despite its known antidepressant-like properties at a dosage of 10 mg/kg, emphasizes the complex nature of its effects on memory processes [46,68,69]. The studies examining sub-anesthetic doses of ketamine in a task designed to evaluate non-spatial recognition memory (the novel object recognition test) discovered that ketamine interfered with the consolidation process [48,70]. Nonetheless, evidence indicates that although Ketamine may affect memory acquisition and retrieval of spatial memory in rats, its impact on the consolidation is not substantial [48,71]. We acknowledge as a limitation of our study that the assessment of the cognitive effects resulting from the combined administration of an NPY1R agonist and Ketamine was confined to a single memory test. Future investigations would benefit from incorporating a broader range of cognitive tests to comprehensively evaluate the impact of these treatments on various aspects of memory and learning.

In the current study, we chose naive rat models as an essential preliminary step to assess the fundamental effects of the NPY1R agonist and ketamine combination on cognitive functions and neuronal growth. This methodological choice is grounded in the demonstrated importance of naive rat models for unraveling the pathophysiological mechanisms underlying mood disorders and assessing the prophylactic potential of therapeutic strategies. Our approach is aimed at facilitating a comprehensive understanding of neurobiological and behavioral aspects related to neurodegeneration and cognitive decline.

This foundational exploration sets the groundwork for subsequent, more targeted investigations into therapeutic interventions for neurodegenerative conditions. We are committed to incorporating models of neurodegenerative disease in our future research endeavors. The inclusion of these models will allow us to directly assess the therapeutic improvements offered by the NPY1R agonist and ketamine combination in a context that closely mirrors the pathological state of neurodegenerative diseases, thereby enhancing the translational value of our findings.

These forthcoming studies are critical for understanding the broader applicability of these treatments across different models of neurodegeneration and age-related cognitive impairments, enriching our knowledge of their neuropharmacological mechanisms and therapeutic potential. We regard the current results as preliminary and are fully committed to extending our research to include models of neurodegeneration, aligning with our long-term objective of developing effective treatments for cognitive decline associated with neurodegenerative diseases.

Our investigation into the cellular underpinnings of NPY1R agonist and Ketamine co-administration focused on the potential formation of NPY1R-TrkB heteroreceptor complexes within the dorsal DG. Although our findings indicate receptor co-localization, the definitive proof of heteroreceptor complex formation and their functional interplay requires further elucidation through advanced methodologies, including Co-Immunoprecipitation (CoIP), Fluorescence Resonance Energy Transfer (FRET), and Bioluminescence Resonance Energy Transfer (BRET). These methods provide sensitive and specific means to detect and characterize the dynamics of protein interactions within the cellular environment. Previous research on GALR2/NPY1R heteroreceptor complexes supports the possibility of allosteric modulation enhancing NPY1R-TrkB signaling, which could underlie the observed memory consolidation improvements [42,45,53,56,58,60,61].

The notable increase in PCNA-immunoreactive cells and specifically DCX-expressing neuroblasts, without a corresponding rise in GFAP-expressing cells, suggests that the drug combination selectively stimulates neuroblast proliferation. This is consistent with findings that NPY enhances cell proliferation in the dorsal DG under neurodegenerative conditions, but not in a healthy hippocampus [39]. Similarly, while Ketamine’s effects on adult hippocampal neurogenesis remain ambiguous, evidence suggests it does not promote neural progenitor proliferation or differentiation into DCX-positive cells [72,73].

Moreover, the proliferative effects observed with the NPY1R agonist and Ketamine treatment appear to be mediated by the increased expression of BDNF in the dorsal hippocampus. The relationship between BDNF, its TrkB receptor, neurodegenerative disorders, and neuroprotection is well documented, with diminished BDNF levels observed in various neurodegenerative diseases and correlating with disease severity [74,75]. Enhancing BDNF pharmacologically has shown potential in ameliorating Alzheimer’s disease pathology and memory impairment, emphasizing BDNF’s essential role in adult hippocampal neurogenesis and cognitive function maintenance [76,77].

This research introduces an innovative perspective on the synergistic effects of the NPY1R agonist and Ketamine, potentially mediated by the formation of NPY1R-TrkB heteroreceptor complexes, highlighting a novel pathway for the enhancement in cognitive functions and neuronal growth. Such mechanisms offer a hopeful perspective for therapeutic strategies targeting neurodegenerative diseases and cognitive impairments, advocating for further in-depth investigations into these interactions and their clinical implications.

## 5. Conclusions

Our research provides foundational insights into the synergistic effects of NPY1R agonist and Ketamine administration on enhancing memory consolidation and stimulating neuroblast proliferation within the dorsal hippocampus. These effects, observed in a healthy rat model, suggest a complex interplay between NPY1R and Ketamine pathways in modulating neuroplasticity and cognitive functions, potentially mediated by the formation of NPY1R-TrkB heteroreceptor complexes.

The observed increase in PCNA-immunoreactive cells and specifically in DCX-expressing neuroblasts, alongside the mediation of these effects by BDNF expression, points to the critical role of BDNF in neuronal growth and cognitive function. While our study offers a preliminary exploration of these compounds’ neuropharmacological effects, the translation of our findings to therapeutic strategies for neurodegenerative conditions necessitates further investigation.

In sum, this study highlights the potential of targeting NPY1R and Ketamine pathways as a novel approach for cognitive and neuronal enhancement. Future research should aim to validate these findings in neurodegenerative disease models, thereby expanding our understanding of their therapeutic value and mechanisms of action.

## Data Availability

The data that support the findings of this study are openly available in the institutional repository of the University of Málaga (RIUMA) and from the corresponding author upon reasonable request.

## Supplementary Materials

The following supporting information can be downloaded at: https://www.mdpi.com/article/10.3390/cells13080669/s1, Table S1: Total exploration time on training and test sessions in the object-in-place memory task.
