# Supplementary material for: Enhancing Cognitive Functions and Neuronal Growth through NPY1R Agonist and Ketamine Co-Administration: Evidence for NPY1R-TrkB Heteroreceptor Complexes in Rats
Source: Cells. 2024 Apr 12;13(8):669. doi: 10.3390/cells13080669 (PMC11049095; doi:10.3390/cells13080669)
Supplement: Supplementary file 1 [file cells-13-00669-s001.zip › cells-2928421-supplementary.pdf]

Supplementary Material

Enhancing Cognitive Functions and Neuronal Growth through NPY1R Agonist and Ketamine Co-administration: Evidence for NPY1R-TrkB Heteroreceptor Complexes in Rats

Carlos Arrabal-Gómez 1,2,3,4; Rasiel Beltran-Casanueva 5,6; Aracelis Hernández-García 5,6; Juan Vicente Bayolo-Guanche 5,6; Miguel Angel Barbancho-Fernández 1; Pedro Serrano-Castro 1,3,4* and Manuel Narváez 1,3,4*.

1 NeuronLab, Facultad de Medicina, Instituto de Investigación Biomédica de Málaga, Universidad de Málaga., Málaga, 29071, Spain, carrabal@uma.es; Pedro.serrano.c@gmail.com;mnarvaez@uma.es

2 Facultad de Psicología, Instituto de Investigación Biomédica de Málaga, Universidad de Málaga., Málaga, 29071, Spain,

3 Unit of Neurology, Hospital Regional Universitario de Málaga, Instituto de Investigación Biomédica de Málaga, Málaga, 29010, Spain

4 Vithas Málaga, Vithas Málaga. Grupo Hospitalario Vithas, Málaga, 29016, Spain,

5 Department of Neuroscience, Karolinska Institutet, Stockholm, 17177, Sweden. Email: rasiel.beltran@stud.ki.se. Aracelishernandezgarcia81@gmail.com; Bayolo1969@gmail.com

6 Receptomics and Brain disorders lab, Edificio Lopez-Peñalver, Instituto de Investigación Biomédica de Málaga, Facultad de Medicina, Universidad de Málaga, 29071, Spain.

*Correspondence: mnarvaez@uma.es (MN)

Intranasal Peptide Delivery Method

Peptides involved were sourced from Tocris Bioscience (Bristol, UK). These compounds were freshly prepared by dissolving in 20 µl of distilled water immediately before administration. For the delivery, each rat was administered a total of 10 µl of solution, evenly divided between each nostril (5 µl per nostril). The application was carried out using a pipetteman equipped with a disposable plastic tip, which was carefully inserted into the nostril to a depth of no more than 1–1.5 mm. This procedure was conducted under mild isoflurane anesthesia to ensure minimal discomfort to the animals. Immediately after the infusion, to maximize retention of the solution and enhance absorption, the head of the rat was gently held in an elevated position, tilted backward for about 15 seconds. This precautionary step was crucial to prevent the administered solution from leaking out of the nares, thereby ensuring optimal delivery of the peptides to the target area within the nasal cavity. This intranasal administration method offers a non-invasive, efficient route for delivering therapeutic agents directly to the brain, bypassing the blood-brain barrier and potentially reducing systemic side effects associated with other delivery methods.

Counting Procedure

PCNA and BDNF-labeled cells were counted with an Olympus BX51 microscope, Olympus, Denmark interfaced with a computer and a colour JVC digital video camera. For stereological analysis, sampling of positive cells was performed throughout the dentate gyrus of the dorsal hippocampus in the rostrocaudal dimension using the optical fractionator, according to Paxinos & Watson atlas coordinates [1]. This method combines the optical dissector with a fractionator sampling scheme to exclude volume divergences [2]. Counterstaining with phase contrast allowed delineation of different areas in each section [1]. Numbers of positive cells were quantified in at least five representative 150 µm, evenly spaced sections per animal (4 rats per group). A random set of sampling frames with a known area (α frame) was generated for each section using the C.A.S.T.

Grid (Olympus; Albertslund, Denmark). After the objects were counted (ΣQ-) the total number of positive cells were estimated as: N = ΣQ- x fs x fa x fh (Gundersen et al., 1988), where fs is the numerical fraction of the section used, fa is the areal fraction and fh is the linear fraction of section thickness. The coefficient of error (CE) for each estimation and animal ranged from 0.05 to 0.1. The total CE of each group ranged from 0.07 to 0.08. Counting of labelled cells was set starting at 5 µm below the surface and focusing through the 20 µm section optical plane, and the number of counting frames used was 90-110 per animal. We have used this stereological procedure is previous studies [3-7].

Group	Control	NPY1R Agonist	Ketamine	NPY1R+Ketamine	Y1R+Ketamine+BIBP3226	Y1R+Ketamine +ANA-12
Training (Total exploration time (s))	19.6±0.5	20.2±0.6	19.8±0.7	20.5±1.1	20.6±0.9	21.3±0.9
Test (Total exploration time (s))	19.4±1	20.1±1.1	18.4±1	21.2±0.6	20.2.3±1.2	19.8±0.7

Supplementary table 1. Total exploration time on training and test sessions in the object-in-place memory task.

References

1. Paxinos, G.; Watson, C. *The rat brain in stereotaxic coordinates: hard cover edition*; Elsevier: 2006.
2. Gundersen, H.J.; Bagger, P.; Bendtsen, T.F.; Evans, S.M.; Korbo, L.; Marcussen, N.; Moller, A.; Nielsen, K.; Nyengaard, J.R.; Pakkenberg, B.; et al. The new stereological tools: disector, fractionator, nucleator and point sampled intercepts and their use in pathological research and diagnosis. *APMIS* **1988**, *96*, 857-881, doi:10.1111/j.1699-0463.1988.tb00954.x.
3. Narvaez, M.; Millon, C.; Borroto-Escuela, D.; Flores-Burgess, A.; Santin, L.; Parrado, C.; Gago, B.; Puigcerver, A.; Fuxe, K.; Narvaez, J.A.; et al. Galanin receptor 2-neuropeptide Y Y1 receptor interactions in the amygdala lead to increased anxiolytic actions. *Brain Struct Funct* **2015**, *220*, 2289-2301, doi:10.1007/s00429-014-0788-7.
4. Narvaez, M.; Borroto-Escuela, D.O.; Santin, L.; Millon, C.; Gago, B.; Flores-Burgess, A.; Barbancho, M.A.; Perez de la Mora, M.; Narvaez, J.; Diaz-Cabiale, Z.; et al. A Novel Integrative Mechanism in Anxiolytic Behavior Induced by Galanin 2/Neuropeptide Y Y1 Receptor Interactions on Medial Paracapsular Intercalated

Amygdala in Rats. *Front Cell Neurosci* **2018**, *12*, 119, doi:10.3389/fncel.2018.00119.

5. Mirchandani-Duque, M.; Barbancho, M.A.; Lopez-Salas, A.; Alvarez-Contino, J.E.; Garcia-Casares, N.; Fuxe, K.; Borroto-Escuela, D.O.; Narvaez, M. Galanin and Neuropeptide Y Interaction Enhances Proliferation of Granule Precursor Cells and Expression of Neuroprotective Factors in the Rat Hippocampus with Consequent Augmented Spatial Memory. *Biomedicines* **2022**, *10*, doi:10.3390/biomedicines10061297.
6. Narvaez, M.; Borroto-Escuela, D.O.; Millon, C.; Gago, B.; Flores-Burgess, A.; Santin, L.; Fuxe, K.; Narvaez, J.A.; Diaz-Cabiale, Z. Galanin receptor 2-neuropeptide Y Y1 receptor interactions in the dentate gyrus are related with antidepressant-like effects. *Brain Struct Funct* **2016**, *221*, 4129-4139, doi:10.1007/s00429-015-1153-1.
7. Borroto-Escuela, D.O.; Fores, R.; Pita, M.; Barbancho, M.A.; Zamorano-Gonzalez, P.; Casares, N.G.; Fuxe, K.; Narvaez, M. Intranasal Delivery of Galanin 2 and Neuropeptide Y1 Agonists Enhanced Spatial Memory Performance and Neuronal Precursor Cells Proliferation in the Dorsal Hippocampus in Rats. *Front Pharmacol* **2022**, *13*, 820210, doi:10.3389/fphar.2022.820210.
